# Search for Specific Biomarkers of IFNβ Bioactivity in Patients with Multiple Sclerosis

**DOI:** 10.1371/journal.pone.0023634

**Published:** 2011-08-23

**Authors:** Sunny Malhotra, Marta F. Bustamante, Francisco Pérez-Miralles, Jordi Rio, Mari Carmen Ruiz de Villa, Esteban Vegas, Lara Nonell, Florian Deisenhammer, Nicolás Fissolo, Ramil N. Nurtdinov, Xavier Montalban, Manuel Comabella

**Affiliations:** 1 Centre d'Esclerosi Múltiple de Catalunya, CEM-Cat, Unitat de Neuroimmunologia Clínica, Hospital Universitari Vall d′Hebron (HUVH), Barcelona, Spain; 2 Departament d′Estadística, Facultat de Biologia, Universitat de Barcelona, Barcelona, Spain; 3 Department of Neurology, Innsbruck Medical University, Innsbruck, Austria; Charité-Universitätsmedizin Berlin, Germany

## Abstract

Myxovirus A (MxA), a protein encoded by the *MX1* gene with antiviral activity, has proven to be a sensitive measure of IFNβ bioactivity in multiple sclerosis (MS). However, the use of MxA as a biomarker of IFNβ bioactivity has been criticized for the lack of evidence of its role on disease pathogenesis and the clinical response to IFNβ. Here, we aimed to identify specific biomarkers of IFNβ bioactivity in order to compare their gene expression induction by type I IFNs with the MxA, and to investigate their potential role in MS pathogenesis. Gene expression microarrays were performed in PBMC from MS patients who developed neutralizing antibodies (NAB) to IFNβ at 12 and/or 24 months of treatment and patients who remained NAB negative. Nine genes followed patterns in gene expression over time similar to the *MX1*, which was considered the gold standard gene, and were selected for further experiments: *IFI6*, *IFI27*, *IFI44L*, *IFIT1*, *HERC5*, *LY6E*, *RSAD2*, *SIGLEC1*, and *USP18*. In vitro experiments in PBMC from healthy controls revealed specific induction of selected biomarkers by IFNβ but not IFNγ, and several markers, in particular *USP18* and *HERC5*, were shown to be significantly induced at lower IFNβ concentrations and more selective than the *MX1* as biomarkers of IFNβ bioactivity. In addition, *USP18* expression was deficient in MS patients compared with healthy controls (p = 0.0004). We propose specific biomarkers that may be considered in addition to the MxA to evaluate IFNβ bioactivity, and to further explore their implication in MS pathogenesis.

## Introduction

In 1993, IFNβ became the first FDA-approved drug for the treatment of relapsing-remitting MS (RRMS), and since then it has widely been used in clinical practice. IFNβ has demonstrated beneficial effects on decreasing the number of clinical relapses and disease activity measured by magnetic resonance imaging [1]–[3]. The mechanisms of action by which IFNβ produces its therapeutic effects in MS are not yet fully understood, however, IFNβ beneficial effects are most likely associated with its immunomodulatory properties.

IFNβ is a type I IFN that binds a heterodimeric cell surface receptor composed of the IFN receptor 1 (IFNAR1) and 2 (IFNAR2) subunits and activates the JAK-STAT signaling pathway. As a result, IFN-stimulated gene factor 3 (ISGF3) complexes are formed and translocated to the nucleus where they bind to IFN-stimulated response elements (ISREs) and initiate the transcription of type I IFN-responsive genes [4]. Among the different type I IFN-responsive genes, myxovirus resistance protein A (MxA), a GTPase protein encoded by the *MX1* gene with potent antiviral activity [5], has proven to be one of the most sensitive and specific biomarkers of IFNβ bioactivity [6], [7]. MxA expression is significantly reduced during the development of neutralizing antibodies (NABs) [8]–[10], and its measurement has provided the basis for in vitro and in vivo assays to determine the presence of NABs [11], [12]. However, there is a lack of clear roles of MxA as a biomarker on disease pathogenesis or in the therapeutic response to IFNβ.

In the present study, we aimed to identify new biomarkers of IFNβ bioactivity in order to compare their specificities as genes induced by type I IFNs with the MxA, and evaluate their potential implication in MS pathogenesis.

## Results

### Microarray studies identify biomarkers of IFNβ bioactivity with similar gene expression patterns to the *MX1*

We first performed gene expression microarrays in PBMC collected at different time points from IFNβ-treated patients. Supplementary Tables S1 and S2 show the top canonical pathways that were identified in up- and down-regulated genes respectively during IFNβ treatment compared to the baseline condition. As expected, the type I IFN signaling pathway was one of the most significant pathways identified among up-regulated genes.

**Figure 1:**
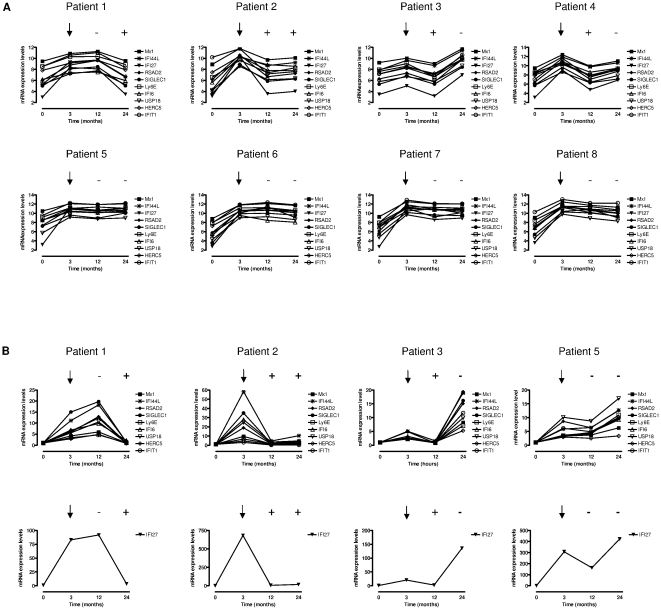
(A) Changes in gene expression observed with microarrays for selected IFNβ bioactivity markers at baseline (T = 0), and after 3, 12, and 24 months of treatment. Four patients developed NABs at 12 and/or 24 months (Patients 1–4) and 4 patients remained NAB negative at these time points (Patients 5–8). (B) Validation of microarray findings by real time RT-PCR in representative patients belonging to the different categories (Patients 1, 2, 3, and 5). Given the much stronger induction in gene expression observed for *IFI27*, graphs corresponding to its expression were depicted separately for the sake of clarity. Open squares: *Ly6E*; open circles: *IFIT1*; open triangles: *IFI6*; open inverted triangles: *USP18*; open diamonds: *HERC5*; asterisks: *IFI44L*; solid squares: *MX1*; solid circles: *SIGLEC1*; solid inverted triangles: *IFI27*; solid diamonds: *RSAD2*. ↓: refers to induction in gene expression after 3 months of treatment. +: NAB positive determination. -: NAB negative determination.

In order to identify new markers of IFNβ bioactivity, we stratified patients based on the presence and absence of NABs at 12 and/or 24 months of IFNβ treatment. Nine genes fulfilled the conditions described in the Methods section and followed patterns of gene expression over time similar to the *MX1*, the gold standard gene, and were chosen for further experiments (Table 1). As shown in Figure 1A, selected genes were significantly induced by IFNβ treatment after 3 months of treatment and their expression levels were reduced by the presence of NABs and reversed in NAB negative conditions.

**Table 1:** Selected markers of IFNβ bioactivity from gene expression microarrays.

Affymetrix probe set	Symbol	Description	Other aliases and designations	Chromosome location
202086_at	*MX1* *	myxovirus (influenza virus) resistance 1, interferon-inducible protein p78 (mouse)	MxA	21q22.3
204415_at	*IFI6*	interferon, alpha-inducible protein 6	IFI-6-16, G1P3	1p35
202411_at	*IFI27*	interferon, alpha-inducible protein 27	ISG12	14q32
204439_at	*IFI44L*	interferon-induced protein 44-like	C1orf29	1p31.1
203153_at	*IFIT1*	interferon-induced protein with tetratricopeptide repeats 1	IFI56, ISG56	10q25-q26
219863_at	*HERC5*	hect domain and RLD 5	CEB1, CEBP1	4q22.1
202145_at	*LY6E*	lymphocyte antigen 6 complex, locus E	RIGE	8q24.3
213797_at/242625_at	*RSAD2*	radical S-adenosyl methionine domain containing 2	viperin	2p25.2
219519_s_at	*SIGLEC1*	sialic acid binding Ig-like lectin 1, sialoadhesin	CD169	20p13
219211_at	*USP18*	ubiquitin specific peptidase 18	ISG43	22q11.21

*MX1 was used as the gold standard gene.

Further analysis of potential transcription factors binding to the promoter region of selected genes revealed that seven of them (*MX1*, *IFI27*, *IFIT1*, *RSAD2*, *USP18*, *IFI44L*, and *HERC5*) had ISRE responding elements (STAT1 transcription factor binding sites) in upstream regions very close to the annotated transcription initiation sites, findings that support the specificity of selected biomarkers as type I IFN induced genes.

We next performed real time RT-PCR of selected genes in order to validate microarray findings. As depicted in Figure 1B, mRNA expression levels measured by PCR over time in NAB positive and negative patients mirrored those obtained with gene expression microarrays.

### Selected IFNβ bioactivity markers are specifically induced by type I IFNs

**Figure 2:**
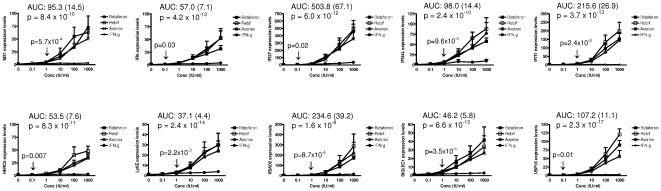
Dose-dependent induction in gene expression of selected IFNβ bioactivity biomarkers. PBMC from 6 healthy controls were cultured for 24 hours with Avonex (asterisks), Rebif (open squares), Betaferon (solid squares), and recombinant IFNγ (solid circles) at different concentrations (Conc; x-axis). After cell culture, mRNA expression levels were determined by real time RT-PCR, as described in Methods. Results are expressed as fold change in gene expression relative to the untreated condition (0 IU/ml). Bars represent SEM. AUC: area under the curve (SEM) of the difference between IFNβ and IFNγ inductions, together with the associated p-value (selectivity). Arrows indicate the p-values resulting from the comparisons in gene expression between the different IFNβ concentrations and the untreated conditions (lower limit of quantification).

As a next step, we performed in vitro experiments to characterize the specific induction of selected biomarkers by type I (IFNβ) but not type II (IFNγ) IFNs. First, we cultured PBMC from healthy controls for 8 hours in the presence or absence of different concentrations of Avonex, Rebif, Betaferon, and IFNγ. As shown in Figure 2, all genes were selectively induced by IFNβ, as indicated by the differences in gene expression observed for IFNβ and IFNγ. The different types of IFNβ resulted in similar levels of gene expression and were considered together for calculations. Four genes had a lower limit of quantification (LLOQ) of 0.1 IU/ml: *HERC5* (p = 0.007), *USP18* (p = 0.01), *IFI27* (p = 0.02), and *IFI6* (p = 0.03)(Figure 2, arrows). The remaining genes, included *MX1*, reached statistical significance in their gene expression inductions at higher IFNβ concentrations (LLOQ: 1 IU/ml). Except for *RSAD2*, all the selected biomarkers were shown to be more selective than the *MX1* gene, as indicated by the p-values associated with the area under the curve (AUC) of the difference between IFNβ and IFNγ. *USP18* had the lowest p-value (p = 2.3×10^−17^) and was considered to be the most selective IFNβ biomarker. Four genes (*IFI27*, *IFIT1*, *RSAD2*, and *USP18*) had stronger inductions in gene expression by IFNβ compared with the *MX1*, whereas *IFI6*, *IFI44L*, *HERC5* and *SIGLEC1* showed gene expression levels comparable to the *MX1*. Finally, *Ly6E* was up-regulated at lower levels (Figure 2).

From these dose-dependent experiments, a concentration of 100 IU/ml was considered optimal for gene expression induction and selected for further experiments.

**Figure 3:**
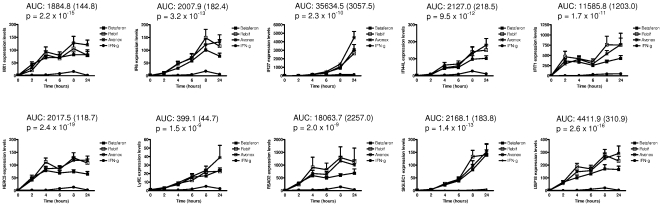
Time-dependent induction in gene expression of selected IFNβ bioactivity markers. PBMC from 7 healthy controls were cultured at different time points in the presence or absence of 100 IU/ml of Avonex (asterisks), Rebif (open squares), Betaferon (solid squares), and recombinant IFNγ (solid circles). At each time point, mRNA expression levels for each gene were determined by real time RT-PCR, as described under Methods. Results are expressed as fold change in gene expression relative to the uncultured condition (0 h) after subtraction of the expression levels obtained for untreated cells. Bars represent SEM. AUC: area under the curve (SEM) of the difference between IFNβ and IFNγ inductions, together with the associated p-value (selectivity).

Next, we cultured PBMC from healthy controls at different time points with 100 IU/ml of IFNβ and IFNγ. As depicted in Figure 3, comparisons of the AUC obtained for gene expression at the different time points revealed *HERC5* (p = 2.4×10^−19^) and *USP18* (p = 2.6×10^−16^) as the genes showing the highest differences in their expression levels between IFNβ and IFNγ. The remaining genes showed lower selectivity values compared with the *MX1* (p = 2.2×10^−15^). Similar to the dose-dependent induction, *IFI27*, *IFIT1*, *RSAD2*, and *USP18* were more up-regulated at the different time points by IFNβ than the *MX1*. On the other hand, *IFI6*, *IFI44L*, *HERC5*, and *SIGLEC1* showed comparable levels of gene expression induction to the *MX1*, whereas *Ly6E* was the least induced gene at all time points (Figure 3).

For most of the biomarkers, peak levels of gene expression occurred after 8 hours of cell culture and this time point was chosen for further experiments.

These data indicate that, although all the selected genes are specifically induced by type I but not type II IFNs, several biomarkers appear to be induced at lower IFNβ concentrations and more selective than the *MX1*.

### Gene expression of selected biomarkers is gradually inhibited by increasing NAB titres

We next evaluated the capacity of high and low NAB titres to inhibit the expression of selected IFNβ bioactivity biomarkers. As depicted in Supplementary Figure S1, all biomarkers showed similar profiles of gene expression inhibition by different NAB dilutions, and gene expression was greatly reduced by high NAB titres (undiluted serum and serum dilutions ranging from 1∶3 to 1∶27). At lower NAB titres (1∶81 serum dilutions), except for *SIGLEC1* gene expression of selected biomarkers was reduced by more than 50% of the expression levels obtained for the positive control. At 1∶243 serum dilutions, except for *SIGLEC1*, *IFI44L*, and *Ly6E* gene expression of the remaining biomarkers was reduced by greater than 25% of the positive control expression levels. Interestingly, *RSAD2* showed the highest degree of inhibition in gene expression by low NAB titres, and was the only IFNβ bioactivity biomarker whose expression was reduced by more than 25% of the positive control condition at the highest serum dilutions (1∶729), and greater than 50% after 1∶243 dilutions (Supplementary Figure S1).

Despite similar levels of NAB-induced gene expression inhibition observed for selected biomarkers, these results point to *RSAD2* as the most sensitive biomarker to capture the blocking effect of low NAB titres.

### Abrogation of gene expression of selected biomarkers following cell activation

**Figure 4:**
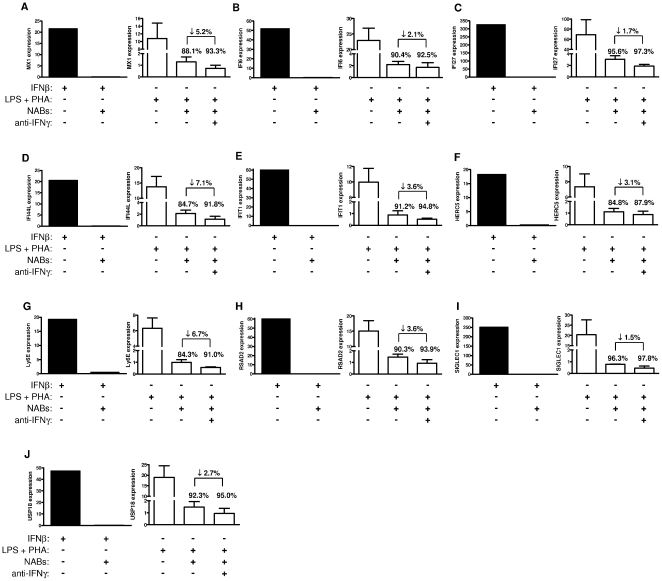
Abrogation of gene expression of selected biomarkers following non-specific cell activation (A–J). PBMC from 3 healthy controls were cultured for 8 hours in preincubated medium with PHA plus LPS in the presence or absence of undiluted high-titre NAB positive serum with and without anti-IFNγ antibodies, as described in Methods. Results are expressed as fold change in gene expression relative to a condition of unstimulated cells and with a value of 1 after normalization (not shown in the graphs for the sake of clarity). PBMC cultured with 100 IU/ml of Betaferon in the presence or absence of high-titre NAB positive serum were used as positive controls (graphs on the left). Bars represent SEM. Arrows indicate the difference in gene expression observed after the addition of anti-IFNγ antibodies. NAB: neutralizing antibodies to IFNβ.

To evaluate whether selected biomarkers could be indirectly induced via the production of cytokines other than IFNβ, PBMC from healthy controls were non-specifically activated with LPS plus PHA in the presence or absence of a high-titre NAB positive serum. As shown in Figure 4, IFNβ accounted for the majority of gene expression induced by non-specific cell activation, as IFNβ blocking was associated with a more than 80% reduction in the expression levels for *MX1*, *IFI44L*, *HERC5*, and *Ly6E*, and greater than 90% reduction for *IFI6*, *IFI27*, *IFIT1*, *RSDA2*, *SIGLEC1*, and *USP18*. As expected from dose- and time-dependent experiments, IFNγ contributed little to cell activation-induced gene expression, and IFNγ blocking only resulted in a small additional decrease in gene expression that ranged from 1.5% for *SIGLEC1* to 7.1% for *IFI44L* (Figure 4).

These findings indicate that cell activation-induced up-regulation of selected biomarkers is mostly mediated by the effects of IFNβ, and other cytokines included IFNγ appear to contribute little to their expression.

### *USP18* expression is deficient in MS patients

**Figure 5:**
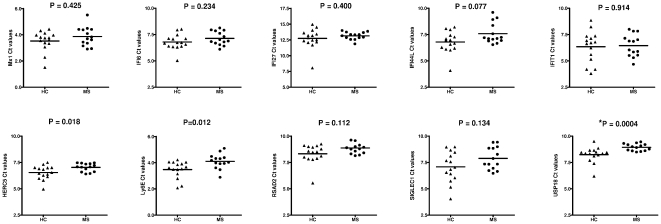
Comparison of gene expression levels of selected biomarkers in MS patients and controls. PBMC were collected from untreated RRMS patients (N = 14) and healthy controls (N = 15) and the mRNA expression levels for each gene were determined by real time RT-PCR. The y-axis represents the threshold cycle (C_T_) values obtained for each individual. C_T_ is inversely related to quantity, and higher C_T_ values are indicative of lower mRNA expression levels. MS: untreated RRMS patients. HC: healthy controls.

We finally aimed to evaluate the potential implication of selected biomarkers in MS pathogenesis. To achieve this, expression levels for these biomarkers were compared between untreated RRMS patients and healthy controls. Interestingly, only *USP18* survived correction for multiple testing, and expression levels for this gene were significantly lower in MS patients compared with controls (p = 0.0004)(Figure 5). Trends towards lower expression in MS patients were also observed for *HERC5* (p = 0.018) and *Ly6E* (p = 0.012), although differences did not reach the threshold for statistical significance after Bonferroni correction (alpha = 0.005). Expression levels for the remaining genes were similar between MS patients and healthy controls (Figure 5).

No significant correlations were observed between USP18 expression levels and variables such as gender, age at onset, EDSS scores at the time of blood collection, number of relapses in the 2 previous years, and disease duration (p>0.05).

## Discussion

MxA is specifically induced by type I IFNs and has demonstrated to be a reliable and sensitive measure of the biological response to IFNβ [6], [7]. However, it has no confirmed roles in MS pathogenesis or in the clinical response to IFNβ. By applying gene expression microarrays to PBMC from patients who developed NABs to IFNβ and patients who remained NAB negative, we identified 9 biomarkers that followed changes in gene expression over time similar to the *MX1*, the gold standard gene. While some of these biomarkers have been used in previous studies to evaluate the biological response to IFNβ [13], [14] (Supplementary Table S3), others have not been tested yet. In the present study, we compared the potential for selected biomarkers to evaluate IFNβ bioactivity. Interestingly, although *MX1* induction was highly selective for type I IFNs, dose- and time-dependent induction experiments revealed several biomarkers of IFNβ bioactivity that were more selective, and significantly induced by lower IFNβ concentrations and at higher levels than the *MX1*. The finding of similar profiles of gene expression inhibition by different NAB dilutions for all selected biomarkers supports their use to measure the in vivo effects of NABs on IFNβ bioactivity. Finally, the gene expression abrogation experiments following non-specific stimulation indicate that cytokines other than IFNβ contribute little to the expression of selected biomarkers and reinforce their specificity by type I IFNs. Although not proven in the present study, the low gene expression levels that remained after inhibiting the effects of both IFNβ and IFNγ were most likely due to the action of IFNα, another type I IFN.

*USP18* was one of the most selective biomarkers of IFNβ bioactivity, and was significantly induced at the lowest IFNβ concentration and up-regulated to a greater degree by type I IFNs compared to the *MX1* gene. Furthermore, it was the only biomarker found to be differentially expressed between MS patients and controls, which suggests that *USP18* may play a role in the pathogenesis of MS. *USP18* codes for a type I IFN-inducible cysteine protease that deconjugates ISG15, a ubiquitin-like protein, from target proteins [15]. Interestingly, USP18 has been shown to negatively regulate the type I IFN signalling pathway, and its deficiency results in enhanced and prolonged STAT1 phosphorylation [15]–[17]. This action appears to be independent of its protease activity and mediated by the specific binding of USP18 to IFNAR2, which then blocks the interaction between JAK1 and the IFN receptor and results in inhibition of the downstream phosphorylation cascade [18]. Although further studies are needed, it is tempting to speculate that a deficient expression of *USP18* in MS patients may lead to overactivation of the type I IFN pathway and have implications in the therapeutic response to IFNβ. In fact, overexpression of type I IFN-responsive genes has been associated with a decrease biological and clinical response to IFNβ in MS patients [19], [20]. Whether or not responders and non-responders to IFNβ differ in their allelic frequencies for *USP18* is an open question.

Together with *USP18*, *HERC5* was highly selective as IFNβ bioactivity biomarker, significantly induced at the lowest IFNβ concentration, and showed induction levels comparable to the *MX1*. *HERC5* codes for a protein ligase that is involved in the ISG15 conjugation process (ISGylation) upon stimulation with type I IFNs [21].

Similar to the *MX1*, *RSAD2* was significantly induced at a concentration of IFNβ of 1 IU/ml, but showed stronger induction in gene expression by type I IFNs although with lower selectivity. *RSAD2* (also known as viperin) encodes an antiviral protein that is involved in innate immunity against the infection of many DNA and RNA viruses. Of note, *RSAD2* showed the highest degree of inhibition in gene expression by high dilutions of serum from a NAB positive patient. The inhibiting effect of high and low NAB titres on *RSDA2* expression was also evaluated in a recent study [14]. These findings suggest that RSAD2 measurement may be considered in the design of new and more sensitive assays to determine NABs.

Similar to the *MX1, SIGLEC1* and *Ly6E* had a LLOQ of 1 IU/ml. In dose-dependent induction experiments, they were shown to be more selective than the *MX1* as IFNβ bioactivity biomarkers. However, *Ly6E* induction levels were the lowest following stimulation with IFNβ, and *SIGLEC1* was the least sensitive biomarker to capture the blocking effect of low NAB titres. *SIGLEC1* (also known as CD169) codes for a macrophage-restricted sialic acid receptor, which mediates adhesive interactions with lymphoid and myeloid cells [22]. Although little is known on the function of Ly6E, Ly6 proteins may be playing roles in cell signalling and cell adhesion processes [23], [24]. Interestingly, *SIGLEC1* and *Ly6E* were found to be up-regulated in peripheral blood cells, mainly monocytes, from patients with other autoimmune disorders such as systemic sclerosis and systemic lupus erythematosus compared with healthy controls [25]–[28], and mRNA and protein levels were shown to correlate with disease activity in lupus patients [25], [27], [28]. Studies correlating *SIGLEC1* and *Ly6E* levels with disease activity or the response to IFNβ have not been performed in MS.

In dose-dependent induction experiments, *IFI6* and *IFI27* were significantly induced at lower IFNβ concentrations and more selective than the *MX1*. While *IFI6* showed comparable induction levels to the *MX1*, *IFI27* was by far the most up-regulated gene following stimulation with type I IFNs. Of note, *IFI27* was proposed as a sensitive marker of IFNβ bioactivity in a recent study [13], and in a one-year time course transcriptomic study *IFI6* was found among the genes consistently up-regulated by IFNβ [29]. IFI6 and IFI27 belong to the FAM14 family of proteins and have roles in the regulation of apoptosis. *IFI6* encodes an anti-apoptotic protein that inhibits depolarization of mitochondrial membrane potential, cytochrome c release, and caspase-3 activity [30]. Interestingly, IFI6 has also been shown to antagonize the effects of TRAIL (TNF-related apoptosis-inducing ligand) by inhibiting the intrinsic apoptotic pathway through mitochondrial stabilization [31]. The protein encoded by *IFI27* associates with or inserts into the mitochondrial membrane, and its up-regulation has been reported to lead to decreased viable cell numbers and enhanced sensitivity to DNA-damage induced apoptosis [32]. Given the important role that apoptosis plays in the pathogenesis of MS, further studies to explore the implication of IFI6 and IFI27 in disease pathogenesis are warranted.

Finally, LLOQ of *IFIT1* and *IFI44L* was the same as the *MX1* (1 IU/ml). Whereas in the dose-dependent experiments *IFI44L* showed similar selectivity and induction levels to the *MX1*, *IFIT1* appeared to be more selective and induced to a higher degree compared to the *MX1*. *IFIT1* encodes a protein that is rapidly induced in response not only to viral infections but also non-viral stimuli such as LPS, IL-1 and TNFα [33], [34], and may be involved in cell apoptosis via interaction with the eukaryotic elongation factor-1A (eEF1A)[35]. Little evidence exists in the literature regarding the function of the protein encoded by *IFI44L*. However, it is important to mention that a related gene, *IFI44*, and *IFIT1* were found to be among the genes that best predicted the response to IFNβ treatment in MS patients [19].

In summary, we propose specific biomarkers that may be considered in addition to the MxA to measure the biological response to IFNβ and the in vivo effects of NABs. Although more studies are needed, findings from the present study suggest that some of these selected biomarkers may also be playing roles in the pathogenesis of MS and/or the therapeutic response to IFNβ.

## Materials and Methods

### Ethics Statement

The study was approved by the Hospital Universitari Vall dHebron Ethics Committee [PR(AG)33/2008] and all patients gave written informed consent to be included in the study.

### Gene expression microarrays

PBMC from RRMS patients were collected before and during IFNβ treatment and stored in liquid nitrogen until used. Gene expression microarrays (Affymetrix Human Genome U133 Plus 2.0) were performed in PBMC from 8 RRMS patients at baseline and after 3, 12 and 24 months of IFNβ treatment. All patients were females and the mean age (SD) was 43.1 years (8.8). Four patients were treated with subcutaneous IFNβ -1b (Betaferon), and the remaining received subcutaneous IFNβ-1a (Rebif). Four patients were negative for NABs at 12 and 24 months and 4 patients developed NABs at 12 and/or 24 months (one patient was NAB positive at 12 and 24 months, another patient was negative at 12 and positive at 24 months, and 2 patients were positive at 12 and negative at 24 months).

Quality control, preprocessing and analysis of microarray data were performed as previously described [19]. We aimed to identify genes that followed temporal expression patterns similar to the *MX1*, which was chosen as our ‘gold standard’ gene. To achieve this purpose, graphics of *MX1* gene expression (202086_at affy ID) over time were generated for the 8 patients included in the study, and searched for genes that followed the same pattern in gene expression. First, for each patient, behaviour of *MX1* was analyzed at each time point and determined whether *MX1* gene expression decreased or increased in each time interval: 0–3 months, 3–12 months, and 12–24 months. Next, genes that followed the same increase-decrease pattern in gene expression to the *MX1* were selected. The final list of genes was generated with all common genes in the 8 study patients. The absolute value of change in gene expression was set at 0.8, because 0.83 was the minimum increase in gene expression observed for *MX1* in one of the patients from baseline to the 3 months time point. Pathway analysis was performed with Ingenuity Pathway Analysis (Ingenuity Systems, version 9.0 www.ingenuity.com) using two separate lists of genes, on one side the 816 unique transcripts of up-regulated genes and, on the other side, the 329 unique transcripts list of down-regulated genes. Search of potential binding sites for transcription factors in promoter regions of selected genes was performed using the TRANSFAC database [36] (see Supplementary Methods S1 for a more detailed description of this type of analysis). Microarray data are stored in the Gene Expression Omnibus (GEO) repository and are available at http://www.ncbi.nlm.nih.gov/geo/with the following entry number: GSE26104.

NABs were determined in serum samples at baseline and after 12 and 24 months of treatment by means of the myxovirus A induction bioassay, as described elsewhere [12], and titers equal or higher than 20 neutralizing units were deemed positive results.

### Validation of selected IFNβ bioactivity markers by real time RT-PCR

In 4 patients, expression levels of selected genes were also determined by real time RT-PCR in order to validate microarray findings. Total RNA was taken from the same samples that had been used for the microarrays. cDNA was synthesized from total RNA using the High Capacity cDNA Archive Kit (Applied Biosystems, Foster City, CA, U.S.A). Amplifications were performed in duplicate using Taqman probes specific for the genes selected from microarray studies (Applied Biosystems). The housekeeping gene GAPDH was used as an endogenous control. The threshold cycle (C_T_) value for each reaction, and the relative level of gene expression for each sample were calculated using the 2^−ΔΔCT^ method [37]. In brief, GAPDH was employed for the normalization of the quantity of RNA used. Its C_T_ value was subtracted from that of the specific genes to obtain a **Δ**CT value. The differences (**ΔΔ**CT) between the **Δ**CT values obtained for the untreated baseline samples (calibrators) and the **Δ**CT values for the IFNβ-treated samples (at 3, 12 and 24 months) were determined. The relative quantitative value for the treated samples was then expressed as 2^−ΔΔCT^, representing the fold change in gene expression normalized to the endogenous control and relative to the calibrators.

### Dose- and time-dependent induction of selected IFNβ bioactivity markers

For dose-dependent experiments, fresh PBMC from 6 healthy controls [3 females/3 males; mean age: 27.5 years (7.1)] were isolated by Ficoll-Isopaque density gradient centrifugation (Gibco BRL, Life Technologies LTD, UK), washed twice and resuspended in culture medium (RPMI medium 1640 supplemented with 10% fetal bovine serum, 4 mM L-glutamine, 25 mM Hepes buffer, 50 units/ml penicillin, and 50 µg/ml streptomycin (Gibco BRL). PBMC (2×10^6^ cells/ml) were cultured for 24 hours with intramuscular IFNβ-1a (Avonex), Rebif, Betaferon, and human recombinant IFNγ at different concentrations: 0.1, 10, 100, and 1000 IU/ml. After cell culture, mRNA expression levels of selected IFNβ bioactivity markers were determined by real time RT-PCR, as previously described. Changes in gene expression were always compared with cells cultured in the absence of IFNβ (referred to as 0 IU/ml; calibrators).

For time-dependent experiments, freshly isolated PBMC from 7 healthy controls [3 females/4 males; mean age: 27.5 years (5.7)] were cultured as previously described in the presence or absence of 100 IU/ml of Avonex, Rebif, Betaferon, and human recombinant IFNγ for 2, 4, 6, 8, and 24 hours. After cell culture, gene expression levels for selected markers were determined by real time RT-PCR, as described above. Changes in gene expression were always referred to a baseline uncultured condition (0 h; calibrators). Previously, gene expression levels obtained for the different biomarkers in untreated cells cultured for the same time points were subtracted from the values obtained after treatment with IFNβ and IFNγ.

### NAB-induced gene expression inhibition

Undiluted and serially diluted serum (1∶3, 1∶9, 1∶27, 1∶81, 1∶243, 1∶729) collected from a patient treated with Betaferon who developed NABs at high titres (>1280) was preincubated for 1 hour in the presence or absence of 100 IU/ml of Betaferon. Subsequently, freshly isolated PBMC from 3 healthy controls [2 females/1 male; mean age: 24.7 years (2.1)] were cultured for 8 hours with preincubated medium. After cell culture, mRNA expression levels of selected IFNβ bioactivity markers were determined by real time RT-PCR, as described above. IFNβ-induced expression levels were compared with those obtained from cells cultured without IFNβ in the presence of serum from a NAB negative patient (calibrators). PBMC cultured with 100 IU/ml of Betaferon was used as positive control.

### Abrogation of gene expression induced by non-specific cell activation

Freshly isolated PBMC from 3 healthy controls [2 females/1 male; mean age: 29.3 years (4.9)] were cultured for 8 hours in preincubated medium with PHA (5 µg/ml) plus LPS (1 µg/ml) in the presence or absence of undiluted high-titre NAB positive serum (>1280) with and without anti-IFNγ antibodies (100 ng/ml) at 37°C for 1 hour. After cell culture, gene expression of selected biomarkers was determined by real time RT-PCR, as previously described. Cell activation-induced expression levels were compared with those obtained from unstimulated cells cultured in the presence of serum from a NAB negative patient (calibrators). PBMC cultured with 100 IU/ml of Betaferon in the presence or absence of high-titre NAB positive serum were used as positive controls of NAB-induced inhibition.

### Gene expression levels for selected bioactivity markers in MS patients and controls

Fresh PBMC were isolated from 14 untreated RRMS patients [64.3% females; mean age (standard deviation): 42.1 years (9.6); mean number of relapses in the previous 2 years: 0.9 (0.9); mean disease duration: 12.4 years (7.1); median EDSS at the time of blood collection (interquartile range): 2.0 (1.5–3.0)]. A group of 15 healthy controls [53.3% females; mean age: 30.5 years (6.2)] was also included in the study.

After RNA extraction, mRNA expression levels for selected biomarkers were determined by real time RT-PCR, as described above. Gene expression values obtained for MS patients were referred to the expression levels observed in controls (calibrators).

### Statistical analysis

For dose-dependent experiments, the following parameters were considered: (i) Sensitivity was evaluated by the LLOQ and defined as the minimum IFNβ concentration that induced a statistically significant increase in gene expression when compared with the untreated condition, and it was calculated by paired t-tests adjusting for multiple testing using the Bonferroni approach. (ii) Selectivity was defined, for each gene, as the difference observed in gene expression between different concentrations of type I and type II IFNs, and it was calculated by comparing the AUC obtained for IFNβ and IFNγ. The p-value associated with the AUC of the difference was calculated by means of a t-type statistic that uses the critical value from a t-distribution with Satterthwaite's approximation [38] to the degrees of freedom for calculation of confidence intervals.

Similarly, for time-dependent selectivity was defined as the difference observed in gene expression between type I and type II IFNs at the different time points of in vitro cell culture, and it was analyzed by computing the AUC of the difference, as described above.

NAB-induced gene expression inhibition was evaluated by comparing the NAB-positive serum dilutions that were associated with reductions in gene expression of selected biomarkers greater than 25% and 50% of the expression levels obtained for the positive control condition.

A Mann-Whitneýs test was used to test for significant differences in gene expression levels between MS patients and controls. Insomuch as 10 different genes were evaluated, Bonferroni correction was used to correct the alpha level for multiple comparisons (alpha = 0.005).

Statistical calculations were performed with R language and the SPSS 11.5 package (SPSS Inc, Chicago, IL) for MS-Windows.

## Supporting Information

Figure S1 NAB-induced gene expression inhibition of selected biomarkers. Undiluted and increasingly diluted serum from an IFNβ-treated patient who developed high NAB titres were preincubated for 1 hour in the presence or absence of 100 IU/ml of Betaferon, and then added to PBMC from 3 healthy controls for 8 hours, as described in Methods. Results are expressed as fold change in gene expression relative to a condition of cells cultured without IFNβ and with a value of 1 after normalization (not shown in the graphs for the sake of clarity). Bars represent SEM. Dotted lines indicate the expression levels that correspond to 25% and 50% reductions in gene expression of the positive control condition. US: undiluted serum. PC: positive control. NAB: neutralizing antibodies to IFNβ.
(TIF)

Table S1 Top canonical pathways up-regulated during treatment with IFNβ.
(DOC)

Table S2 Top canonical pathways down-regulated during treatment with IFNβ.
(DOC)

Table S3 Summary of studies related with selected IFNβ bioactivity markers.
(DOC)

Methods S1 (DOC)
