# Supplementary material for: Search for Specific Biomarkers of IFNβ Bioactivity in Patients with Multiple Sclerosis
Source: PLoS One. 2011 Aug 23;6(8):e23634. doi: 10.1371/journal.pone.0023634 (PMC3160307; doi:10.1371/journal.pone.0023634)
Supplement: Table S1 — Top canonical pathways up-regulated during treatment with IFNβ. (DOC) [file pone.0023634.s002.doc]

**Supplementary Table 1.** Top canonical pathways up-regulated during treatment with IFNβ

| **Pathways** | **P-value** | **Ratio*** |
| --- | --- | --- |
| Molecular Mechanisms of Cancer | 0.0016 | 0.0603 |
| Interferon Signaling | 0.0037 | 0.147 |
| Role of Pattern Recognition Receptors in Recognition of Bacteria and Viruses | 0.0102 | 0.0854 |
| Starch and Sucrose Metabolism | 0.0110 | 0.0882 |
| Activation of IRF by Cytosolic Pattern Recognition Receptors | 0.0138 | 0.0923 |
| ERK5 Signaling | 0.0138 | 0.0952 |
| Role of NANOG in Mammalian Embryonic Stem Cell Pluripotency | 0.0263 | 0.0702 |
| Notch Signaling | 0.0324 | 0.093 |
| Sphingolipid Metabolism | 0.0331 | 0.0759 |
| PPARα/RXRα Activation | 0.0355 | 0.0575 |
| T Cell Receptor Signaling | 0.0355 | 0.068 |
| Protein Ubiquitination Pathway | 0.0380 | 0.0519 |
| Glioma Invasiveness Signaling | 0.0389 | 0.0847 |
| Cyclins and Cell Cycle Regulation | 0.0417 | 0.0682 |
| Galactose Metabolism | 0.0417 | 0.0889 |
| Purine Metabolism | 0.0427 | 0.053 |
| Colorectal Cancer Metastasis Signaling | 0.0427 | 0.0526 |
| Role of JAK1, JAK2 and TYK2 in Interferon Signaling | 0.0437 | 0.115 |
| Role of Macrophages, Fibroblasts and Endothelial Cells in Rheumatoid Arthritis | 0.0457 | 0.0464 |
| Role of PI3K/AKT Signaling in the Pathogenesis of Influenza | 0.0479 | 0.0714 |
| Role of JAK family kinases in IL-6-type Cytokine Signaling | 0.0479 | 0.115 |

*Refers to the ratio between the number of genes found up-regulated at any time point during IFNβ treatment compared with the untreated condition and the number of genes belonging to the corresponding pathway.
