# Supplementary material for: Search for Specific Biomarkers of IFNβ Bioactivity in Patients with Multiple Sclerosis
Source: PLoS One. 2011 Aug 23;6(8):e23634. doi: 10.1371/journal.pone.0023634 (PMC3160307; doi:10.1371/journal.pone.0023634)
Supplement: Table S2 — Top canonical pathways down-regulated during treatment with IFNβ. (DOC) [file pone.0023634.s003.doc]

**Supplementary Table 2.** Top canonical pathways down-regulated during treatment with IFNβ

| **Pathways** | **P-value** | **Ratio*** |
| --- | --- | --- |
| IL-8 Signaling | 0.0002 | 0.0559 |
| PI3K/AKT Signaling | 0.0002 | 0.0602 |
| Apoptosis Signaling | 0.0003 | 0.0745 |
| PPAR Signaling | 0.0003 | 0.0673 |
| IL-17A Signaling in Gastric Cells | 0.0004 | 0.1600 |
| Hepatic Fibrosis / Hepatic Stellate Cell Activation | 0.0008 | 0.0563 |
| Ceramide Signaling | 0.0009 | 0.0732 |
| Prostate Cancer Signaling | 0.0010 | 0.0652 |
| Role of IL-17A in Arthritis | 0.0010 | 0.0833 |
| CXCR4 Signaling | 0.0015 | 0.0500 |
| CDK5 Signaling | 0.0016 | 0.0674 |
| PAK Signaling | 0.0017 | 0.0571 |
| Granzyme A Signaling | 0.0017 | 0.1670 |
| Role of NFAT in Regulation of the Immune Response | 0.0022 | 0.0423 |
| HMGB1 Signaling | 0.0022 | 0.0612 |
| IL-6 Signaling | 0.0023 | 0.0612 |
| Angiopoietin Signaling | 0.0024 | 0.0704 |
| Melanoma Signaling | 0.0030 | 0.0909 |
| Protein Kinase A Signaling | 0.0036 | 0.0351 |
| IL-17 Signaling | 0.0038 | 0.0676 |
| ILK Signaling | 0.0040 | 0.0428 |
| Glucocorticoid Receptor Signaling | 0.0040 | 0.0357 |
| fMLP Signaling in Neutrophils | 0.0045 | 0.0508 |

| **Pathways** | **P-values** | **Ratio*** |
| --- | --- | --- |
| Thrombin Signaling | 0.0054 | 0.0408 |
| Airway Pathology in Chronic Obstructive Pulmonary Disease | 0.0055 | 0.2500 |
| Colorectal Cancer Metastasis Signaling | 0.0063 | 0.0364 |
| Regulation of Actin-based Motility by Rho | 0.0066 | 0.0568 |
| Gα12/13 Signaling | 0.0066 | 0.0480 |
| Integrin Signaling | 0.0066 | 0.0385 |
| TNFR2 Signaling | 0.0074 | 0.0909 |
| Glioma Invasiveness Signaling | 0.0091 | 0.0678 |
| SAPK/JNK Signaling | 0.0095 | 0.0495 |
| Acute Phase Response Signaling | 0.0102 | 0.0407 |
| Role of MAPK Signaling in the Pathogenesis of Influenza | 0.0107 | 0.0645 |
| Relaxin Signaling | 0.0117 | 0.0414 |
| Hypoxia Signaling in the Cardiovascular System | 0.0120 | 0.0588 |
| Oncostatin M Signaling | 0.0126 | 0.0882 |
| IL-17A Signaling in Fibroblasts | 0.0138 | 0.0769 |
| Phospholipase C Signaling | 0.0141 | 0.0324 |
| Role of IL-17A in Psoriasis | 0.0145 | 0.1540 |
| Agrin Interactions at Neuromuscular Junction | 0.0151 | 0.0588 |
| ERK/MAPK Signaling | 0.0155 | 0.0354 |
| Erythropoietin Signaling | 0.0158 | 0.0541 |
| B Cell Receptor Signaling | 0.0162 | 0.0400 |

| **Pathways** | **P-values** | **Ratio*** |
| --- | --- | --- |
| April Mediated Signaling | 0.0170 | 0.0714 |
| Role of Tissue Factor in Cancer | 0.0178 | 0.0450 |
| PTEN Signaling | 0.0178 | 0.0413 |
| IL-12 Signaling and Production in Macrophages | 0.0186 | 0.0435 |
| Renin-Angiotensin Signaling | 0.0191 | 0.0446 |
| RhoA Signaling | 0.0191 | 0.0459 |
| Role of PKR in Interferon Induction and Antiviral Response | 0.0195 | 0.0667 |
| Role of IL-17F in Allergic Inflammatory Airway Diseases | 0.0195 | 0.0667 |
| NF-κB Activation by Viruses | 0.0200 | 0.0506 |
| LPS-stimulated MAPK Signaling | 0.0209 | 0.0506 |
| Communication between Innate and Adaptive Immune Cells | 0.0209 | 0.0388 |
| Role of Hypercytokinemia /hyperchemokinemia in the pathogenesis of Influenza | 0.0209 | 0.0698 |
| Acute Myeloid Leukemia Signaling | 0.0240 | 0.0494 |
| Regulation of IL-2 Expression in Activated and Anergic T Lymphocytes | 0.0240 | 0.0471 |
| Germ Cell-Sertoli Cell Junction Signaling | 0.0240 | 0.0373 |
| Altered T Cell and B Cell Signaling in Rheumatoid Arthritis | 0.0251 | 0.0440 |
| PPARα/RXRα Activation | 0.0263 | 0.0345 |
| Differential Regulation of Cytokine Production in Macrophages and T Helper Cells by IL-17A and IL-17F | 0.0269 | 0.1110 |

| **Pathways** | **P-values** | **Ratio*** |
| --- | --- | --- |
| NF-κB Signaling | 0.0275 | 0.0349 |
| p70S6K Signaling | 0.0275 | 0.0403 |
| Actin Cytoskeleton Signaling | 0.0282 | 0.0302 |
| TNFR1 Signaling | 0.0302 | 0.0577 |
| GNRH Signaling | 0.0324 | 0.0376 |
| PI3K Signaling in B Lymphocytes | 0.0324 | 0.0370 |
| Melanocyte Development and Pigmentation Signaling | 0.0331 | 0.0460 |
| Neuregulin Signaling | 0.0339 | 0.0412 |
| VEGF Signaling | 0.0339 | 0.0435 |
| α-Adrenergic Signaling | 0.0355 | 0.0435 |
| Bladder Cancer Signaling | 0.0355 | 0.0440 |
| Ephrin Receptor Signaling | 0.0363 | 0.0309 |
| TREM1 Signaling | 0.0372 | 0.0526 |
| G Beta Gamma Signaling | 0.0380 | 0.0396 |
| Cardiac Hypertrophy Signaling | 0.0380 | 0.0303 |
| Role of Cytokines in Mediating Communication between Immune Cells | 0.0389 | 0.0556 |
| Endometrial Cancer Signaling | 0.0389 | 0.0545 |
| Differential Regulation of Cytokine Production in Intestinal Epithelial Cells by IL-17A and IL-17F | 0.0398 | 0.0909 |
| IL-1 Signaling | 0.0427 | 0.0396 |
| Chronic Myeloid Leukemia Signaling | 0.0437 | 0.0388 |

*Refers to the ratio between the number of genes found down-regulated at any time point during IFNβ treatment compared with the untreated condition and the number of genes belonging to the corresponding pathway.
