# Supplementary material for: Search for Specific Biomarkers of IFNβ Bioactivity in Patients with Multiple Sclerosis
Source: PLoS One. 2011 Aug 23;6(8):e23634. doi: 10.1371/journal.pone.0023634 (PMC3160307; doi:10.1371/journal.pone.0023634)
Supplement: Table S3 — Summary of studies related with selected IFNβ bioactivity markers. (DOC) [file pone.0023634.s004.doc]

Supplementary Table 3. Summary of studies related with selected IFNβ bioactivity markers

| Gene | Reference | Comment |
| --- | --- | --- |
| *IFI6* | Serrano-Fernández P et al. (2010) [29] | Found to be differentially expressed at all time points in a one-year time course transcriptomic study with IFNβ |
| *IFI27* | Sellebjerg et al. (2009) [13] | Considered a sensitive biomarker of response to IFNβ treatment |
| *IFI44L /*  *HERC5* | Comabella et al. (2009) [19] | Differentially expressed between responders and non-responders to IFNβ following comparisons in gene expression between 3 months and baseline time points |
| *IFIT1* | Comabella et al. (2009) [19] | Identified as one of the genes that best predicted the response to IFNβ treatment in MS patients |
| *LY6E* | Feng et al. (2006) [25]; Tang et al. (2008) [28] | Found up-regulated in patients with other autoimmune disorders characterized by a type I IFN signature such as systemic lupus erythematosus |
| *RSAD2* | Pachner et al. (2009) [14] | Patients positive for neutralizing antibodies (NAB) showed lower RSAD2 responses compared with NAB negative patients |
| *SIGLEC1* | York et al. (2007) [26];  Biesen et al. (2008) [27] | Found up-regulated in patients with other autoimmune disorders characterized by a type I IFN signature such as systemic lupus erythematosus and systemic sclerosis |
| *USP18* | Sellebjerg et al. (2009) [13] | Identified as one of the genes induced early after administration of IFNβ |
