# Supplementary material for: Search for Specific Biomarkers of IFNβ Bioactivity in Patients with Multiple Sclerosis
Source: PLoS One. 2011 Aug 23;6(8):e23634. doi: 10.1371/journal.pone.0023634 (PMC3160307; doi:10.1371/journal.pone.0023634)
Supplement: Methods S1 — (DOC) [file pone.0023634.s005.doc]

**Supplementary Methods.**

**Search of potential binding sites for transcription factors in selected genes.**

We searched potential binding sites for STAT transcription factors in promoter regions of selected IFNβ-induced genes. For this, we downloaded corresponding matrixes from the TRANSFAC database [36] (public release 7.0 2005). Unfortunately, matrixes were of poor quality and therefore we decided to use Transcription Factor ChIP-seq from ENCODE track in UCSC genome browser as an additional control.

We downloaded five matrixes for STAT factors from TRANSFAC database (public release 7.0 2005). M00223 matrix was designed to recognize all STATs binding sites, M00224 matrix was designed to recognize only STAT1 factor, M00225 matrix was designed to recognize STAT3. Finally, M00459 and M00460 matrixes were designed to recognize STAT5B homodimer and STAT5A homotetramer respectively. We searched candidate sites in 5kb upstream sequences for selected genes.
